# Research progress of peptides discovery and function in resistance to abiotic stress in plant

**DOI:** 10.1007/s44154-025-00220-1

**Published:** 2025-05-23

**Authors:** Yucong Cao, PingFang Yang, Ming Li

**Affiliations:** State Key Laboratory of Biocatalysis and Enzyme Engineering, School of Life Sciences, Hubei University, Wuhan, 430062 China

**Keywords:** Peptides, Peptidomics, Abiotic stress, Plant

## Abstract

Plant peptides play crucial roles in various biological processes, including stress responses. This study investigates the functions of plant peptides in response to different adversity stresses, focusing on drought, salt, high temperature, and other environmental challenges. In drought conditions, specific peptides such as CLE25 and CLE9 were found to regulate stomatal closure and root architecture to enhance the efficiency of water utilization. Salt stress induces the expression of CAPE1 and CEP3, which are involved in ion homeostasis and osmoregulation, thereby contributing to salt tolerance in plants. Heat stress triggers the expression of peptides such as CEL45, which contributes to the heat tolerance of cells. Besides, we have also verified a new class of non-conventional peptides, and a large number of non-conventional peptides have been identified in rice seedlings. Understanding the origin and functions of these peptides presents both challenges and opportunities for developing stress-resistant crops. Future research should focus on elucidating the precise molecular mechanisms of peptide-mediated stress responses and exploring their potential applications in agriculture and biotechnology.

**Supplementary Information:**

The online version contains supplementary material available at 10.1007/s44154-025-00220-1.

## Introduction

**Fig. 1:**
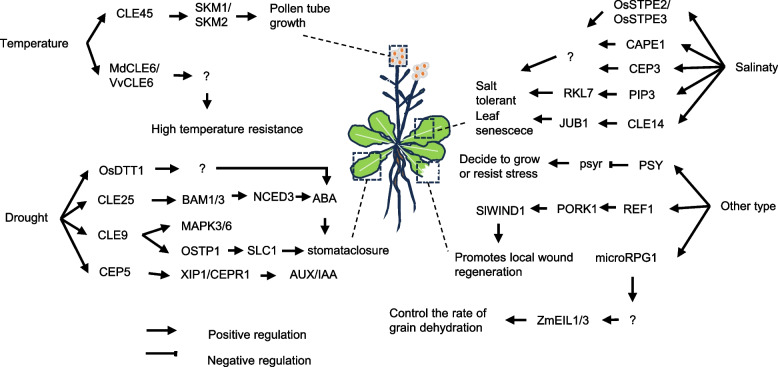
Signaling pathways of plant peptides in response to different stresses

Peptides are typically protein molecules composed of 2–100 amino acid residues (Murphy et al. 2012). Peptides in plants can be simply divided into two categories, namely precursor-derived peptides and non-precursor-derived peptides. Most of the peptides derived from the precursor are encoded by non-functional precursor genes, and the non-functional precursors contain family-specific conserved sequences at the C-terminus (Tavormina et al. 2015). These precursors are cleaved by proteases under specific conditions, and their active forms often require complex post-translational modifications to produce functional small peptide hormones (Matsubayashi 2011). Most mature peptides can bind to the corresponding receptor through the cellular secretion pathway or long-distance infusion, and activate downstream signaling factors such as MAPK (mitogen-activated protein kinase) cascade signaling and transcription factors through phosphorylation, thereby opening up downstream signaling and mediating cell-to-cell communication (Sparks et al. 2013; Haruta et al. 2014; Tabata and Sawa 2014; Butenko and Simon 2015). Due to the specific growth patterns of plants and their frequent exposure to stresses caused by various abiotic factors, this signaling and cell-to-cell communication is important for maintaining normal plant growth. Plants themselves have evolved a plethora of mechanisms to integrate various environmental factors in order to coordinate cellular behavior and overall growth (Zulfiqar et al. 2019; Motte et al. 2019). At present, a large number of studies have shown that small peptides, as an important type of signaling molecule in plants, are widely involved in the transmission of local or long-distance signals in plants and help plants coordinate their tolerance to abiotic stresses (Lay and Takahashi 2018; Gautrat et al. 2021). Bioinformatics methods are currently used to discover gene members of different peptide families from genome sequences. More than 7,000 small peptide-coding genes have been identified in the Arabidopsis genome, most of which may encode hormone-like peptides that play an important role in helping plants resist abiotic stresses such as high temperatures, drought, and high salinity (Takahashi et al. 2019). Systemin, the first bioactive peptide to be identified in plants, contains 18 amino acids from a protein precursor composed of 200 amino acids. Systematics induce the production of protease inhibitors in injured leaves, which will affect the function of the digestive system of insects after ingestion, thereby preventing insects from continuing to invade plants (Pearce et al. 1991). So far, many members of the known CLE(Embryo-surrounding region-related), CEP(C-terminally encoded peptide), PSY(Plant peptides containing tyrosine sulfation) and other families have been confirmed to play an important role in helping plants resist abiotic stress, and the downstream signaling pathways have been analyzed in detail (Mitchum et al. 2008; Delay et al. 2013a; Tost et al. 2021). It shows the great potential of plant peptides in resisting adversity stress. Therefore, we have compiled the latest published research on the role of plant peptides in stress resistance, which provides some insights for further research on the role of small signal peptides in plant stress (Fig. 1).

Compared to peptides derived from precursors, the study of peptides derived from non-precursors is lagging behind, and this type of peptide is directly translated from sORF. The synthesis of these peptides does not require intermediate precursors or further processing (Andrews and Rothnagel 2014). Only a very small number of articles have reported on such peptides. In addition to this, a new class of peptides has attracted a lot of attention in research, and it has been named non-conventional peptides because it is very different from traditional peptides (Ma et al. 2014; Couso and Patraquim 2017; Chen et al. 2020; Jackson et al. 2018). The researchers used a six-frame translation approach for genome-wide data to construct a custom peptide database of maize and Arabidopsis. This database facilitates the large-scale identification of non-conventional peptides in these plants, challenging the traditional understanding of the composition of plant peptides. Notably, the team mapped these peptides to their genomic locations, revealing that a large portion came from non-coding regions, such as the 5' untranslated region, the 3' untranslated region, introns, and intergenic regions. This discovery breaks with previous notions of transcription and translation mechanisms and highlights multiple translational patterns of endogenous plant peptides (Wang et al. 2020). The non-conventional peptides identified by the use of metagenomics are expected to open a new door for the study of the stress resistance of plant peptides, and the proportion of these peptides in plants is even greater than that of traditional peptides. Non-conventional peptides, as a very important class of endogenous peptides in plants, exist in large quantities in plants. The origin and function of these peptides have not yet been revealed in current research (Plaza et al. 2017). In this paper, we review the previously reported small peptides that play an important role in plant stress resistance, and at the same time verify the new non-conventional peptides, which is expected to dig out new clues related to plant stress resistance in the new nonconventional peptides, aiming to promote the research on the role of plant peptides in abiotic stress.

## Biological functions of plant peptides in response to different adversity stresses

### The role of plant peptides in drought resistance

The CLAVATA3 (CLV3) family is a prominent plant peptide group, while the *CLE* (*CLV3/ESR*) gene encoding a large family of peptide molecules in plants (Betsuyaku et al. 2011). Named after its first discovered member, CLAVATA3 in Arabidopsis, the *CLE* gene family consists of 32 genes in the Arabidopsis genome (Hirakawa and Sawa 2019; Song et al. 2021a; Chu et al. 2006). Extensive research has shown that various members of this family play a variety of roles in plant growth, development, and stress responses. They act as key components of cell-to-cell communication, interfering with signal transduction and response pathways in response to adversity stress (Zhang et al. 2020; Bashyal et al. 2024). For instance, CLE25, originally identified in roots, has been found to translocate from roots was transferred from roots to leaves through vascular tissues. This translocation triggers the expression of *nine cis-epoxycarotenoid dioxygenase 3* (*NCED3*), a gene encoding the pivotal enzyme Dioxygenase3 for abscisic acid synthesis (Iuchi et al. 2001; Endo et al. 2008). Consequently, abscisic acid (ABA) accumulation in leaves is enhanced, leading to stomatal closure and improved plant resilience against dehydration stress. The expression of CLE9 in stomata mirrors that of the CLE25 peptide and plays a crucial role in inducing stomatal closure (Takahashi et al. 2018). Research has revealed the presence of the CLE9 peptide in stomata, underscoring its significant involvement in regulating stomatal closure (Jun et al. 2010; Pillitteri et al. 2011). Enhancing induced stomatal closure and drought tolerance in plants can be effectively achieved through the application of the CLE9 peptide or by overexpressing *CLE9*. Conversely, mutants with impaired CLE9 function exhibit heightened susceptibility to drought stress. The induction of stomatal closure by CLE9 is reliant on endogenous ABA and necessitates the presence of two key stomatal signaling components, namely Open Stomata 1 (OST1) and slow anion channel-associated 1 (SLAC1). Moreover, the CLE9 peptide demonstrates the capability to activate MPK3 and MPK6 protein kinases, yet its ability to induce stomal closure is compromised in MPK3 and MPK6 mutants. Activation on of H_2_O_2_ and NO synthases by the CLE9 peptide leads to stomatal closure, a process impeded in mutants deficient in NADPH oxidase-deficient mutants or nitric oxide synthase. The study highlights a novel role of CLE9 in regulating stomatal opening, contingent upon ABA, and underscores the potential function of CLE9 in plants responding to environmental stresses (Wang et al. 2016). The study findings provide new insights into the regulation of stomatal motility in plants through small peptide signaling, paving the way for further investigation into the molecular mechanisms governing plant in responses to environmental stresses like drought. Unlike CLE25, CLE9 is localized in stomata, rather than in vascular tissues. Interestingly, CLE9-mediated stomatal closure operates independently of the BAM/CLV1 receptor, indicating a parallel mechanism to CLE25-stimulated stomatal closure. While the potential for shared downstream signaling components between CLE9 and CLE25-mediated closure exists, ABA is required for both peptides to induce stomatal closure, suggesting a convergence of protective cellular ABA and CLE signaling pathways. In summary, although CLE9 and CLE25 peptides regulate stomatal closure through potentially distinct signaling components, their impact on stomatal closure is partly mediated by their ability to elicit protective cellular ABA signals. The evolutionary rationale behind *Arabidopsis* *thaliana* possessing two parallel CLE signaling pathways to regulate stomatal opening, as well as the utilization of the root CLE25 peptide for rapid stomatal response via long-distance signal transduction influencing ABA biosynthesis, remain unknown.

Recent studies have revealed the involvement of C-terminal encoding peptide 5 (CEP5) in enhancing plant drought resistance, in addition to the well-known CLE family. CEP5, a precursor-derived small peptide, is implicated in various plant developmental processes, particularly in response to abiotic stresses (Delay et al. 2013a; Roberts et al. 2016). Through proteomic and phosphoproteomic analysis of *CEP5*-overexpressing *Arabidopsis* *thaliana* seedlings, researchers observed alterations in processes linked to abiotic stress. The signaling mediated by CEP5 is crucial for enhancing osmotic and drought stress tolerance in Arabidopsis by counteracting the effects of auxin. This signaling mechanism involves the stabilization of AUX/IAA transcriptional repressors, representing a novel peptide-dependent control mechanism for regulating auxin signaling. Overexpression of CEP5 has been found to improve osmotic and drought stress tolerance in plants, potentially through the regulation of CEP5 AUX/IAA stability. The findings underscore the significance of AUX/IAAs in stress tolerance and unveil a novel role for CEP5 in enhancing osmotic and drought stress tolerance. The interplay between auxin and CEP5 appears pivotal in modulating the auxin response threshold and fine-tuning the auxin response for growth and development via the stabilization of AUX/IAAs. CEP5 modulates plant responses to drought and osmotic stress by influencing the expression of auxin response genes, suggesting a novel mechanism for regulating auxin signaling. This study provides fresh insights into the mechanisms by which plants modulate their responses to abiotic stresses through the small peptide CEP5, presenting a potential molecular target for further investigation into plant fitness (Smith et al. 2020). In addition, the small peptide gene *OsDT11* found in rice varieties encodes a cysteine-rich small peptide, and studies have shown that *OsDT11* is involved in regulating drought tolerance in rice. Overexpression of *OsDT11* could significantly increase the content of ABA and reduce plant water evaporation, thereby significantly enhancing the drought tolerance of rice. However, the drought tolerance of plants with *OsDT11* gene knockdown was significantly reduced, and the water loss rate of plants was significantly correlated with the expression of *OsDT11*, which showed that OsDT11 small peptide was an important factor in regulating rice drought resistance (Li et al. 2017).

In summary, small peptides play an important role in enhancing plants resistance to drought. However, the precise activation mechanisms of small peptides in response to changes in water availability or osmotic pressure, as well as the intricate signaling network and subsequent cascading reactions linked to developmental signals, require further elucidation in future research.

### The role of plant peptides in resistance to salt stress

Plants exhibit high sensitivity to elevated salt levels, which can trigger various types of stress including osmotic stress, ionic stress, and oxidative stress (Kamran et al. 2019; Kumar et al. 2013). This can lead to disruptions in cell membrane permeability, nutrient deficiencies, and the accumulation of harmful metabolites, ultimately impeding plant growth (Ahanger and Agarwal 2017). In response to high salt stress, plants have developed defense mechanisms such as stomatal closure to minimize water loss through evapotranspiration, ion transport mechanisms, and the synthesis of compatible solutes to counteract high levels of Na^+ ^(Wu 2018). Additionally, plants possess an extensive antioxidant system to mitigate the effects of salt-induced stress. In plant species, a class of peptides derived from precursor proteins is characterized by a high cysteine content (2–16 Cys) in their mature form, with these cysteines playing a crucial role in peptides structure and function (Marshall et al. 2011; Haag et al. 2012; Vriens et al. 2014). A recent study utilized homology search and mass spectrometry techniques to identify a salt-responsive small peptide, Arabidopsis CAP-derived peptide 1 (AtCAPE1), originating from the pathogenesis-related 1 proteins (CAP) superfamily. The findings revealed that AtCAPE1, a homolog of the tomato immunomodulator CAPE1, is involved in modulating the plant’s response to salt stress, potentially indicating a trade-off between pathogen defense and salt tolerance mechanisms. AtCAPE1 is suggested to regulate salt tolerance by influencing transcription factors within the ABA signaling pathway. The identification of AtCAPE1 as a novel plant peptide in Arabidopsis provides a new perspective on understanding the mechanism by which plants integrate various environmental stresses responses (Chien et al. 2015). The *CLE14* gene, a member of the *CLE* family previously discussed, is implicated in salt stress response. Various environmental cues, such as high salinity, abscisic acid (ABA), salicylic acid, and jasmonic acid, can induce the expression of the *CLE14* gene. Particularly, under high salinity conditions, there is a significant upregulation of *CLE14* gene expression. Knockout of *CLE14* in plants accelerates leaf senescence under salt stress, whereas overexpression of *CLE14* delays this process. These findings suggest that CLE14 modulates plant salt stress tolerance by regulating leaf senescence. Specifically, under salt stress conditions, CLE14 suppresses reactive oxygen species (ROS)-mediated leaf senescence by enhancing the JUB1-mediated ROS scavenging mechanism to mitigate ROS accumulation in leaves. This underscores the crucial role of CLE14 in maintaining redox balance during plant responses to salt stress. Acting as a negative regulator, CLE14 modulates leaf senescence by controlling the JUB1 transcription factor, highlighting its significance in plant adaptation to salt stress (Zhang et al. 2022). In summary, the *CLE14* gene is not only linked to salt stress but also plays a pivotal role in enhancing plant salt stress tolerance through the regulation of leaf senescence and ROS equilibrium.

In addition, it has been found that the expression of *prePIP3* gene, a precursor of small peptide PIP3 (PIMP-Induced secreted Peptide3), was significantly induced by salt stress. The mature PIP3 small peptide will be secreted to the extracellular region, form a complex with RECEPTOR-LIKEKINASE7 (RLK7) under salt stress, and amplify the salt tolerance signal of plants through downstream MPK3 and MPK6 cascade reaction to enhance the salt tolerance of plants (Zhou et al. 2022). In addition, salt stress can also induce the expression of *AtPROPEP3*, a small peptide precursor gene, and Arabidopsis* AtPROPEP3* knockdown transgenic plants showed a highly sensitive phenotype under salt stress. Functional analysis of AtPep3 receptor mutants has shown that AtPep3 can be recognized by its receptor PEPR1 in response to salt stress processes (Nakaminami et al. 2018). The expression of the *CEP* gene is differentially regulated by environmental cues such as salinity. In *Arabidopsis*, CEP3 was significantly upregulated under NaCl treatment (Aggarwal et al. 2020). In addition, some exhibits of Arabidopsis CEP3 mutations are resistant to saline, which is indicated by long variations that are the main root cause in saline therapy. However, the molecular mechanism of CEP3-mediated salt stress response is still unclear (Delay et al. 2013b). In rice, a comparative peptidomic analysis was conducted on rice seedlings exposed to varying concentrations of salt ions. This study identified a substantial number of differentially expressed peptides between the treatment and control group. Candidate peptides associated with salt stress were selected through functional enrichment analysis of the protein precursors of these differentially expressed peptides. Subsequently, three identified candidate peptides were introduced into *Arabidopsis thaliana* through transgenic experiments. The findings demonstrated that salt stress notably enhanced the germination rate and cotyledon greening rate of transgenic *Arabidopsis* *thaliana* expressing *OsSTPE2* and *OsSTPE3* (Ma et al. 2022).This study revealed two novel rice peptides that confer salt tolerance in plants.

### The role of plant peptides in heat stress

Elevated temperatures in plants lead to morphological, anatomical, and biochemical alterations at the tissue level (Li et al. 2018). At high temperatures (more than 40 °C in Arabidopsis), it can severely damage the cells of the plant and quickly lead to cell death. At moderate high temperatures (about 28 ~ 37 °C), the obvious harmful effects on plant growth and reproduction can seriously drill the normal growth of plants. In reaction to heat stress, plants have evolved diverse strategies, which involve upholding membrane stability, synthesizing heat shock proteins (HSPs) and ROS-scavenging enzymes, and triggering chaperone signaling (Zhu et al. 2024; Sakata et al. 2010; Bi et al. 2022). In Arabidopsis in vitro pollen tube culture system, researchers observed that the CLE45 peptide belonging to the CLV3/ESR (CLE) family could extend pollen tube growth. Screening mutants of leucine-rich receptor-like kinase genes in Arabidopsis led to the discovery of two potential receptors for the CLE45 peptide, namely sterility-regulating kinase member 1 (SKM1) and SKM2. The expression of CLE45 is expressed in the stigma of the flower column was found to expand to the pollen tube growth with a temperature increase from 22 °C to 30 °C. The CLE45-SKM1/SKM2 signaling pathway was shown to decrease seed yield at 30 °C but had no impact at 22 °C, as demonstrated by inhibiting of CLE45 expression through RNAi or introducing a kinase-inactivated form of SKM1 in skm1 plants. Additionally, in vitro pollen tube culture experiments revealed that the application of the CLE45 peptide mitigated mitochondrial decay. This signaling pathway involving CLE45-SKM1/SKM2 was found to enhance successful seed production by sustaining pollen viability at elevated temperatures (Endo et al. 2013). In Arabidopsis, AtCLE9, a member of the CLV3/ESR (CLE) peptide family, exhibits a transcriptional response to temperature stress. However, the precise functional significance of CLE9 in plant temperature response remains ambiguous. Furthermore, the functionality of *CLE* genes in various plant species has been partially confirmed. For instance, research indicates that high temperatures can up-regulate the expression of *MdCLE6* in apple and *VvCLE6* in grape (Wang et al. 2019; Ren et al. 2023). Despite these findings, the exact mechanism by which these plant peptides operate remains unclear, necessitating further investigation to elucidate how they enhance plants' heat resistance capabilities.

### Role of plant peptides in other types of adversity stress

In the whole life cycle of plants, in addition to being susceptible to stress caused by environmental changes, they are also very susceptible to various mechanical damage, resulting in damage or loss of some organs or tissues, seriously hindering the growth of plants, and even leading to plant death. Despite this physical damage, plants have developed a strong regenerative capability. For example, plants can regenerate organs or even intact plants from damaged tissues (Birnbaum and Sánchez Alvarado 2008; Mathew and Prasad 2021; Sugimoto et al. 2019). Studies have shown that there is an additional, previously unknown cell-to-cell communication pathway in plants, which can be a trade-off between growth and stress resistance. The mechanism is predominantly based on the plant peptides containing tyrosine sulfation (PSY) family, which consists of a wide range of structurally related tyrosine sulfonate peptides (Ogawa-Ohnishi et al. 2022). In a damaged state, the plant's PSY receptor within the Leucine-rich repeat receptor kinases (LRR-RKs) family is unable to detect the PSY signal. Functioning as a redundant growth inhibitor, PSYR suppresses plant growth, prompting a defensive reaction. This shift in energy allocation enables the plant to prioritize stress resistance and successfully navigate the damaged stage. Upon the plant’s return to normal state, the PSY produced by the organism interacts with the PSYR. The PSY family peptides then inhibit the PSYR signal to maintain optimal growth under regular conditions. Unlike LRR-RKs that typically activate signals upon ligand binding, PSY receptors (PSYRs) induce the expression of various genes encoding stress response transcription factors when ligands are depleted. The absence of PSYRs results in compromised plant tolerance to both biotic and abiotic stresses. This ligand-deprivation-dependent activation mechanism potentially allows plants to regulate stress responses in tissues near the site of metabolic dysfunction, where there is impaired ligand production.

In addition, a recent study showed that there is also a localized system-independent local damage signal in plants, named regenerative factor 1 (REF1) by the authors, as a system-independent local damage signal that mainly regulates local defense responses and regenerative responses in response to damage. Systemin is the first bioactive peptide isolated from plants. However, signal-deficient tomato mutants lack a systemic defense response but still maintain an intermediate local defense response. When the body is damaged, the production of the REF1 ligand, which binds to and activates its receptor PORK1, initiates a *slwind1*-regulated regenerative response. In addition to coordinating cell regeneration reprogramming, activated WOUND-INDUCED DEDIFFERENTIATION 1 (SlWIND1) binds to the vascular-system-specific and wound-responsive *cis*-element (VWRE) motif of the REF1 precursor gene to activate its expression, thereby amplifying REF1 signaling for regenerative responses. This positive feedback loop between REF1-pork1 and SlWIND1 amplifies and stabilizes the REF1 signal. At the same time, REF1 acts as a local wound signal to promote plant regeneration. The discovery of REF1 provides a convenient method to improve the transformation efficiency of stress-tolerant crops by increasing their regenerative ability. In addition, the mode of action of REF1 is similar to that of cytokines in animal immunity and regeneration, suggesting that plants and animals have a similar conceptual logic in regulating trauma-induced tissue repair and organ regeneration. This discovery not only advances our understanding of the mechanism of plant regeneration, but also provides a potential tool for crop genetic improvement. From this perspective, the discovery of REF1 may provide a molecular basis for understanding why plants exhibit higher regenerative capacity than animals (Yang et al. 2024). Through genetics, molecular biology and bioinformatics, the regeneration mechanism of plants after physical injury was deeply studied, which provided an important theoretical basis for future plant regeneration research and crop improvement, and also expanded the diversity of small peptide functions.

At last, we note that a recent study mentions the discovery of a quantitative trait locus (QTL) *qKDR1* in maize, which is a non-coding sequence that regulates the expression of *qKDR1 REGULATED PEPTIDE GENE (RPG)* genes (Yu et al. 2024). The *RPG* gene encodes a 31-amino acid microRPG1 that controls Kernel dehydration rate (KDR) by precisely regulating gene expression in the ethylene signaling pathway. *MicroRPG1* is a new gene endemic to the genus Zea and originates from non-coding sequences. Knockdown of *microRPG1* results in accelerated KDR in maize, while overexpression or exogenously applied micropeptides show the opposite effect. The study also found that microRPG1 has a similar function in Arabidopsis, suggesting that it may have a conserved function in different plant species. This study reveals the molecular mechanism of microRPG1 in regulating maize seed dehydration and provides an important tool for future crop breeding. The authors also explore the molecular mechanism of the origin of new genes, the ability of non-coding sequences to acquire functional micropeptides through single nucleotide mutations. This has led to a new understanding of the origin and function of plant micropeptides Table 1.

**Table 1:** Plant peptides respond to different types of environmental stresses

Type of environment	peptides	receptors	function	References
Drought	CLE25	Atbam1/3	Induce *NCED3* expression, resulting in ABA accumulation in leaves, stomatal closure and reduced water evaporation	(Takahashi et al., 2018)
	CLE9	Atbam1	Drought-induced expression in stomata, resulting in stomatal closure	(Zhang et al., 2019)
	CEP5	XIP1/CEPR1	CEP5 affects osmosis and drought stress tolerance by stabilizing AUX/IAA transcriptional repressors	(Smith et al., 2020)
	OsDTT1		Increase ABA content, reduce water evaporation and improve drought resistance	(Li et al., 2017)
Salinity	CAPE1		AtCAPE1 negatively regulates salt stress tolerance by inhibiting related salt stress genes	(Chien et al., 2015)
	AtCLE14		Salinity-induced accumulation of AtCLE14 slows leaf senescence by controlling the homeostasis of reactive oxygen species	(Endo et al., 2013)
	PIP3	RLK7	The salt tolerance signal of plants was amplified by MPK3 and MPK6 cascades to enhance the salt tolerance of plants	(Zhou et al.,^2022^)
	CEP3		Induced by salt stress, the salt tolerance of plants was improved	(Aggarwal et al., 2020)
	OsSTPE2 /OsSTPE3		Induced by high salt concentration, the salt tolerance ability of rice seedlings was enhanced	(Ma et al., 2022)
Temperature	CLE45	AtSKM1/SKIM2	Pollen tube growth is prolonged at high temperatures	(Endo et al., 2013)
	MdCLE6/ VvCLE6		Expression is induced by high temperatures	(Wang et al. 2019; Zhang et al., 2022)
Other type	PSY	PSYR	As a negative feedback regulation signal to help make trade-offs when the plant is in adversity, the energy is mainly used to resist stress	(Ogawa-Ohnishi et al., 2022)
	REF1	PORK1	REF1 acts as a local wound signaling and promotes plant regeneration by activating the PORK1-SlWIND1 signaling pathway	(Yang et al., 2024)
	microRPG1		Controls kernel dehydration rate by precisely regulating the expression of genes in the ethylene signaling pathway in the kernels after filling	(Yu et al., 2024)

## Challenges and opportunities for plant peptides to cope with adversity research

### Peptide identification and functional research are poised to undergo rapid development

The more we understand the essential functions of small peptides in plants (Birnbaum and Sánchez Alvarado 2008; Mathew and Prasad 2021; Sugimoto et al. 2019; Ogawa-Ohnishi et al. 2022), the greater number of small peptides that will be identified and their functions examined. To advance peptide function research, it is essential to enhance the identification of peptides. Proteomics technology is essential for protein identification and will similarly be vital for peptide identification. With advances in mass spectrometry and the continuous development of bioinformatics, the field of peptidomics has been strengthened, providing valuable methods for identifying new peptides in plants. In fact, peptidomics has indeed shown robust abilities in peptide identification (Wang et al. 2020; Ali et al. 2024). Thousands or even tens of thousands of peptides have been identified in various experiments.

**Fig. 2:**
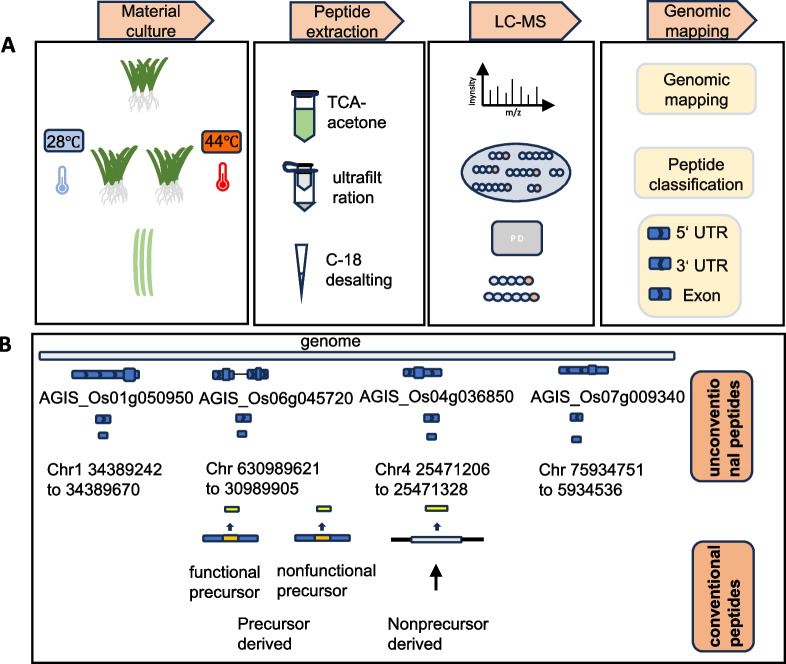
Plant peptidomics workflow and classification of plant peptides. **A** The collected plant samples were extracted from the small peptides by TCA-acetone precipitation method, and the mass spectrometry analysis was performed after desalting in 10 kDa ultrafiltration tubes and C18. Peptide searches were performed using Proteome Discoverer 2.4 to classify peptides from different sources. **B** The existing peptides and the non-conventional peptides identified are briefly classified, and the existing peptides are mainly obtained by the cleavage of the premise protein or the direct translation of the open reading frame. Non-conventional peptides can be identified from introns, intergenic regions, 5' untranslated regions, 3' untranslated regions

In our lab, we refer to the method of peptide genomics and use the six-box translation principle to establish a rice peptide database, trying to find non-conventional peptides related to rice high temperature stress (Fig. 2). Peptides were extracted from seedlings of Nipponbare and Minghui 63 which were planted in normal and high temperature incubator. A total of 572 and 472 peptides were identified from Minghui 63 and Nipponbare at normal growth condition respectively. Similarly, a total of 532 and 417 peptides were identified from Minghui 63 and Nipponbare at high temperature growth condition respectively. We found that the overlapping peptides of the four samples with the existing Coding sequence (CDS) intervals were 39% (221) for CK-MH63, 45% for CK-NIP (213), 43% (231) for HT-MH63, and 44% for HT-NIP (182). The number of nonconventional peptides exceeded that of conventional peptides. In addition to this, the remaining peptides come from introns, 5' untranslated regions, 3' untranslated regions, intergenic regions, and so on. Our results are broadly consistent with previous studies (Wang et al. 2020).

Consequently, the utilization of its advanced mass spectrometry identification capability and comprehensive genomic information, investigating peptidomics in different species is crucial for identifying peptides with potential biological functions. There is no doubt that more results in plant proteomics will be reported in the foreseeable future.

### Challenges encountered in peptidomics and research on peptide function

Despite the progress made in utilizing peptidomics to explore plant peptides, several challenges hinder its practical researches, primarily concerning the following five aspects.

1. The study of plant peptides has only emerged in recent years (Fig. 3). The large-scale identification of plant peptides is still a nascent field, hindered by the abundance of secondary metabolites in plant tissues and the scant presence of peptides. The isolation of peptides proves challenging, On the one hand, there is digestion of a large number of non-specific proteases during sample preparation, which destroys the integrity of the sample peptide group (Secher et al. 2016). Although peptide extraction methods are constantly being updated, many attempts have been made, such as the addition of protease inhibitors to reduce the degradation of proteases during extraction, and microwave radiation binding protease inhibitors. However, past studies have shown that protease inhibitors are limited in preventing peptide degradation (Parkin et al. 2005). In addition, there have been attempts to extract peptides from protoplasts, but the results have not been satisfactory (Luo et al. 2019). This makes it more challenging to isolate intact endogenous peptides in plants, as there are special components in plant cells, such as cell walls, large vacuoles, and chloroplasts, which are more complex than animal cells. Therefore, the non-specific degradation associated with proteases during peptide extraction will be a long-term problem, and there is currently no perfect solution to prevent this from happening. Therefore, more efforts are needed to develop a more efficient peptide extraction protocol that keeps endogenous peptides in the same state in vivo as peptidomics studies. Furthermore, when some possess molecular weights too diminutive for mass spectrometry detection. The successful identification of peptide segments through mass spectrometry is greatly contingent on the extent of small peptide databases, which presently represents a bottleneck in peptidomics technology.

**Fig. 3:**
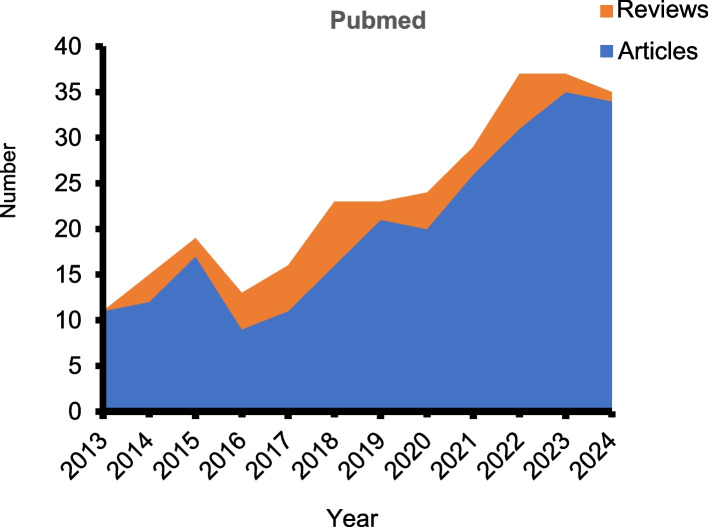
A survey of the literature shows that research on plant peptides has been gradually emerging in the past decade. The data is downloaded from the PubMed database
2. The annotation method for identifying peptide remains undeveloped. Unlike in genome research, where homologous alignment can annotate gene functions, the limited number of peptides with known functions precludes the translation of genomics methods to peptidomics. Consequently, many peptides are identified via mass spectrometry without accurate functional predictions, greatly impeding functional research.
3. Research into peptide modification is at an early stage, as peptides are essentially similar to proteins and may undergo common protein modifications. Yet, the lower abundance peptides presents a more challenging scenario for studying post-translational modifications in comparison to proteins.
4. Studying the interactions among small peptides and other biomolecules is essential. Peptides, categorized as small protein molecules, can engage in interactions with each other, proteins, RNA, and DNA. The establishment and functional analysis of these interaction networks are pivotal for a comprehensive understanding of peptide functions. Nevertheless, many of these studies are still in their nascent stages and await publication.
5. It is essential to delve into efficient methods for conducting functional research on numerous identified plant peptides. Traditional research on plant peptides tends to focus on a single peptide or peptide family, so in general, our understanding of signal peptides in plants is still very limited. Taking the *CLE* gene family as an example, although many *CLE* genes are differentially regulated by environmental stimuli, most *CLE* genes lack experimental verification of their functions (Chu et al. 2006; Bashyal et al. 2024; Wang et al. 2016; Czyzewicz et al. 2015). One of the main reasons for this is due to the small size of *CLE* genes, which are difficult to identify in the genome in addition to the conserved *CLE* domain, and they may be functionally redundant and can collectively regulate certain developmental processes, with multiple potential peptide receptor signaling cascades enabling plants to transmit a wide range of environmental signals under different conditions, with only a few identified peptide or RLKs members having well-defined biological functions (Song et al. 2021b). To date, the biological role of most peptides remains undisclosed and there are shortcomings in rapid exploration methods to elucidate their functions.

In the future, the research on plant peptides needs to be deepened, and the combination of biological problems and cutting-edge technologies in the field of plant peptide research will better expand our understanding of plant peptides. We believe that in the near future, our understanding of plant signal peptides will be clearer, and the functional sources and specific functions of this new type of non-conventional peptide will also be clarified, and the mystery of plant peptides will be gradually revealed.

## Supplementary Information

Supplementary Material 1: Table S1. Detailed information of peptides identified in Sample1 (CK-MH63).

Supplementary Material 2: Table S2. Detailed information of peptides identified in Sample2 (CK-NIP).

Supplementary Material 3: Table S3. Detailed information of peptides identified in Sample3 (L-MH63).

Supplementary Material 4: Table S4. Detailed information of peptides identified in Sample4 (L-NIP).

## Data Availability

Data and materials will be made available on request.

## Abbreviations

MAPK: Mitogen-activated protein kinase
CLV3: CLAVATA3
NCED3: Nine cis-epoxycarotenoid dioxygenase 3
OST1: Open stomata 1
SLAC1: Slow anion channel-associated 1
ABA: Abscisic acid
CEP5: C-terminal encoding peptide 5
AtCAPE1: *Arabidopsis* CAP-derived peptide 1
CAP: *Cysteine*-rich secretory proteins, *antigen* 5, and *pathogenesis*-related 1 proteins
ROS: Reactive oxygen species
SKM1: Sterility-regulating kinase member 1
PSY: Plant peptides containing tyrosine sulfation
LRR-RKs: Leucine-rich repeat receptor kinases
REF1: Regenerative factor 1
WIND1: WOUND-INDUCED DEDIFFERENTIATION 1
VWRE: Vascular-system-specific and wound-responsive cis-element
QTL: Quantitative trait locus
